# Clinical Implications of Urinary C-Peptide Creatinine Ratio in Patients with Different Types of Diabetes

**DOI:** 10.1155/2019/1747684

**Published:** 2019-08-07

**Authors:** Yanai Wang, Ying Gao, Xiaoling Cai, Ling Chen, Lingli Zhou, Yumin Ma, Siqian Gong, Xueyao Han, Linong Ji

**Affiliations:** Departments of Endocrinology and Metabolism, Peking University People's Hospital, Peking University Diabetes Center, Beijing 100044, China

## Abstract

**Introduction:**

Urinary C-peptide creatinine ratio (UCPCR) is used as a marker of endogenous insulin secretion. This study aims to assess the effectiveness of UCPCR for distinguishing between type 1 diabetes (T1DM) and non-T1DM (monogenic diabetes and T2DM) and predicting therapeutic choices in type 2 diabetes (T2DM) patients.

**Methods:**

Twenty-three patients with genetically confirmed monogenic diabetes (median age 35.0 years (interquartile range 30.0-47.0), 13 (56.5%) men), 56 patients with T1DM (median age 46.0 years (interquartile range 26.5-59.5), 28 (50.0%) men), 136 patients with T2DM (median age 53.0 years (interquartile range 42.0-60.0), 87 (64.0%) men), and 59 healthy subjects (median age 36.0 years (30.0-42.0), 26 (44.1%) men) were included. UCPCR was collected in the morning. Receiver operating characteristic (ROC) curves were used to identify optimal UCPCR cut-off values to differentiate T1DM from non-T1DM. This UCPCR cut-off was used to divide T2DM patients into two groups, and the two groups were compared.

**Results:**

The UCPCR was lower in patients with T1DM compared with T2DM, monogenic diabetes, and healthy subjects, while the UCPCR was similar in T2DM and monogenic diabetes. A UCPCR cut-off of ≥0.21 nmol/mmol distinguished between monogenic diabetes and T1DM (area under the curve [AUC], 0.949) with 87% sensitivity and 93% specificity. UCPCR ≥ 0.20 nmol/mmol had 82% sensitivity and 93% specificity for distinguishing between T2DM and T1DM, with an AUC of 0.932. UCPCR was not reliable for distinguishing between monogenic diabetes and T2DM (AUC, 0.605). Twenty-five of 136 (18.4%) T2DM patients had UCPCR ≤ 0.20 nmol/mmol. Compared with T2DM patients with a UCPCR > 0.20 nmol/mmol, T2DM patients with UCPCR ≤ 0.20 nmol/mmol had a lower serum C-peptide (fasting C-peptide, 0.39 nmol/L vs. 0.66 nmol/L, *P* < 0.001; postprandial C-peptide, 0.93 nmol/L vs. 1.55 nmol/L, *P* < 0.001), lower BMI (22.8 kg/m^2^ vs. 25.2 kg/m^2^, *P* = 0.006), and higher percentage of insulin or secretagogue therapy (92.0% vs. 59.5%, *P* = 0.002).

**Conclusions:**

UCPCR is a practical and noninvasive marker that can distinguish between TIDM and T2DM or monogenic diabetes. UCPCR ≤ 0.20 nmol/mmol reflects severe impaired beta cell function and the need for insulin or secretagogue therapy in T2DM patients.

## 1. Introduction

Classification of diabetes remains a challenge for endocrinologists, especially in young patients [1]. Typical clinical symptoms and laboratory tests are the main method of differentiating between types of diabetes. However, with the increase in childhood obesity, the onset age of T2DM is earlier and the number of T1DM patients with either normal weight or overweight has increased. As reported, among newly diagnosed diabetes patients ≤ 19 years old, only two-thirds were T1DM patients in the USA [2]. Therefore, body mass index (BMI) and onset age of diabetes may currently be less specific for distinguishing between T1DM and T2DM [3, 4].

Maturity-onset diabetes of the young (MODY) is the most common monogenic diabetes, accounting for ~2% of all diabetes [5, 6]. The population prevalence of MODY in the United Kingdom is estimated to be 68 to 108 cases per million [7]. MODY can be easily confused with T1DM because of the early onset age of diabetes and lack of obesity. Additionally, simple family history and insulin resistance markers did not reliably distinguish between MODY and T2DM in adults [7–9]. Farmer and Fox reported that only about 15% of young diabetes patients had been correctly diagnosed [10]. Misdiagnosis leads to improper insulin prescription, and it may have a significant impact on quality of life and long-term health outcomes [8].

Serum C-peptide is widely used to assess islet beta cell function, and it is helpful for individualizing treatment in diabetes. The half-life of C-peptide is 20–30 min, which is much longer than that of insulin (half-life, 3–5 min) [11]. C-peptide is mostly metabolized by the kidneys. Urinary C-peptide (UCP) quantity reflects 5–10% of the total C-peptide that is secreted by islet beta cells [12]. Twenty-four-hour UCP was proposed as a noninvasive measure to screen insulin deficiency and as an additional tool to improve clinical classification of diabetes [13]. Because of its time dependence, 24 h UCP is less convenient than spot UCPCR. Fasting UCPCR was shown to be well correlated with 24 h UCP (*r* = 0.8, *P* = 0.00006) [14], which suggests that fasting UCPCR might be an easy method to evaluate C-peptide secretion. Moreover, in the past decade, UCPCR was reported to distinguish between T1DM and non-T1DM (i.e., T2DM and MODY) with a high sensitivity and specificity [15–17]. However, research on UCPCR in diabetes classification has been infrequent in East Asia, and it is not clear whether UCPCR can be used to guide individualized treatment of patients with T2DM.

We aimed to assess whether UCPCR can distinguish between T1DM and monogenic diabetes, including hepatocyte nuclear factor 1-*α* maturity-onset diabetes of the young (MODY3), mitochondrial diabetes (MIDD), and T2DM, and to determine whether UCPCR is a useful tool for predicting therapeutic choices in T2DM patients in a Chinese population.

## 2. Materials and Methods

### 2.1. Subjects

We recruited 56 T1DM patients (fasting serum C-peptide (FCP) < 0.2 nmol/L, ketosis-onset and insulin-dependent treatment within 6 months from onset or adult onset, positive islet autoantibodies, and insulin-dependent insulin treatment), 23 patients with monogenic diabetes (ten patients with HNF1A variants (A311D, p.P353L, R263C, T10M, P379A, R131W, R131W, R200W, ivs8+1, and Q324Term) that induced diabetes (MODY3), 13 patients with mitochondrial A3243G mutation-induced diabetes (MIDD)), and 136 T2DM patients from Peking University People's Hospital, Beijing, China. Type 2 diabetes was defined as adult nonketosis onset diabetes with negative islet autoantibodies (glutamate decarboxylase antibody, insulin antibody, and islet cell antibody).

Diabetes was diagnosed in accordance with the 1999 World Health Organization (WHO) criteria. Patients with monogenic diabetes were included if they had known causative variants and a confirmed molecular genetic diagnosis. The research team used standardized questionnaires to obtain the following information: age, sex, age at diagnosis of diabetes, family history of diabetes, diabetic complications, and current medication. We measured the waistline (WL), blood pressure, weight, and height of every patient. The subjects with an estimated glomerular filtration rate (eGFR) < 60 mL/min/1.73 m^2^ or hypoglycemia during 24 h urine sample collection were excluded.

The healthy subjects were selected based on the following standards: (1) normal blood glucose: no history of diabetes, fasting blood glucose (FBG) < 6.1 mmol/L, 75 g oral glucose tolerance test (OGTT) 2 h < 7.8 mmol/L, and HbA1c (A1C) < 6.0%; (2) blood pressure: lack of hypertension history, systolic pressure (SBP) < 140 mmHg, and diastolic pressure (DBP) < 90 mmHg; (3) BMI < 24 kg/m^2^; (4) WL male < 90 cm and female < 85 cm; (5) normal blood lipids: no history of hyperlipidemia, cholesterol (CHO) < 6.2 mmol/L, triglycerides (TGs) < 1.7 mmol/L, low-density lipoprotein-cholesterol (LDL − C) < 4.1 mmol/L, male high-density lipoprotein-cholesterol (HDL − C) ≥ 0.9 mmol/L, and female HDL − C ≥ 1.0 mmol/L; (6) normal liver function: alanine aminotransferase (ALT) ≤ 50 U/L, aspartate aminotransferase (AST) ≤ 40 U/L; (7) normal renal function: male creatinine < 104 *μ*mol/L and female creatinine < 84 *μ*mol/L; (8) blood uric acid: males < 428 *μ*mol/L and females < 357 *μ*mol/L; (9) urinary albumin/creatinine ratio (ACR) < 30 mg/g; (10) leukocytes > 4 × 10^9^/L; (11) hemoglobin: males ≥ 120 g/L and females ≥ 110 g/L; and (12) no history of hyperuricemia.

Written informed consent was obtained from all subjects. This study was conducted in accordance with the Declaration of Helsinki and was approved by the Ethics Committee at Peking University People's Hospital, Beijing, China.

### 2.2. Sample Collection

Urine and blood samples after 8–12 hours of fasting were collected in the morning. Blood samples at 2 h after breakfast were also collected. The urine samples were stored at −80°C and assayed on the same day. Blood samples were tested on the collecting day.

### 2.3. Laboratory Methods

Urinary C-peptide was measured using an electrochemiluminescence immunoassay on a Roche Diagnostics Cobas e601 analyzer in the endocrine department at the People's Hospital of Peking University, Beijing, China. The lower limit of the C-peptide assay was 0.03 nmol/L. Urinary creatinine was analyzed on the Roche Cobas e311 platform using creatinine Jaffé reagent, and the results were used to calculate UCPCR (nmol/mmol). For the purpose of the analysis, all UCP values < 0.03 nmol/L were coded as 0.03 nmol/L.

### 2.4. Statistical Analysis

Results are presented as the median (interquartile range (IQR)), unless otherwise stated. Characteristics of patients with monogenic diabetes, T1DM, T2DM, or healthy subjects were compared using a chi-squared test for categorical data (e.g., sex, treatment, and parental history), and the Mann–Whitney *U* test for continuous variables for data that were not normally distributed (age at diagnosis, diabetes duration, FCP, postprandial C-peptide (PCP), TG, and UCPCR) were used. Additionally, an independent sample *t*-test was used for the normally distributed variables, and a one-way analysis of variance (ANOVA) followed by the subsequent LSD (Least-Significant Difference) test was used for comparisons between two groups. ROC curves were used to identify cut-off values of UCPCR that provided the optimal sensitivity and specificity (maximizing the Youden index) for distinguishing monogenic diabetes and T2DM from T1DM. *P* < 0.05 was considered to be significant. Statistical software SPSS 16.0 was used for the statistical analysis. Statistical software MedCalc V15.2 was used for comparing different ROC curves of serum C-peptide and UCPCR.

## 3. Results

### 3.1. Clinical Characteristics of the Study Participants

All patient characteristics are presented in Table 1. T1DM patients were younger and slimmer than T2DM, and they received insulin treatment. The onset age of T2DM was older compared with the other two diabetes groups. Parental diabetes was most prevalent in patients with MODY. There were no statistical differences in diabetes duration among diabetes groups.

### 3.2. The Comparison of UCPCR among Different Types of Diabetes

The UCPCR was lower in the T1DM group compared with T2DM (median (IQR), 0.03 (0.01–0.10) nmol/mmol vs. 0.47 (0.23–1.01) nmol/mmol, *P* < 0.001) and monogenic diabetes groups (0.67 (0.26–1.45) nmol/mmol, *P* < 0.001). The UCPCR was similar in the T2DM and monogenic diabetes groups (*P* = 0.099). The UCPCR was higher in healthy subjects (0.71 (0.40–1.08) nmol/mmol) compared with those with T1DM (*P* < 0.001) or T2DM (*P* < 0.001), but it was similar to those with monogenic diabetes.

### 3.3. The Performance of UCPCR in Distinguishing between T1DM and Non-T1DM

**Figure 1:**
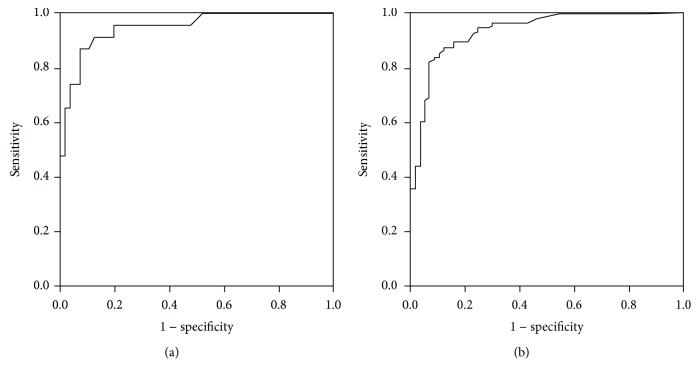
ROC curve to identify T1DM from non-T1DM. (a) The ROC curve identified a cut‐off UCPCR ≥ 0.21 nmol/mmol for discriminating monogenic diabetes from T1DM (AUC 0.949) with 87% sensitivity and 93% specificity. (b) The ROC curve identified a cut‐off UCPCR ≥ 0.20 nmol/mmol for discriminating T2DM from T1DM (AUC 0.932) with 82% sensitivity and 93% specificity.

UCPCR distinguishes between T1DM and monogenic diabetes. UCPCR ≥ 0.21 nmol/mmol had the highest Youden index for identifying monogenic diabetes, with 87% sensitivity and 93% specificity (AUC, 0.949, 95% confidence interval (CI) (0.898–1.000), *P* < 0.001; Figure 1). UCPCR did not reliably distinguish between monogenic diabetes and type 2 diabetes (AUC, 0.605, 95% CI (0.474–0.736), *P* = 0.107). UCPCR ≥ 0.20 nmol/mmol distinguished between T2DM and T1DM with 82% sensitivity and 93% specificity (AUC, 0.932, 95% CI (0.893–0.971), *P* < 0.001; Figure 1).

We compared the ROC curves between UCPCR and serum C-peptide in distinguishing T1DM and non-T1DM by the software MedCalc V15.2 (difference of areas under the curve between serum C-peptide and UCPCR was 0.0619, *Z* statistic was 0.348, and *P* = 0.7279).

### 3.4. The Clinical Characteristics of T2DM Patients with UCPCR ≤ 0.2 nmol/mmol

The characteristics of T2DM patients are presented in Table 2. Twenty-five of 136 (18.4%) T2DM patients had UCPCR ≤ 0.20 nmol/mmol. Compared with T2DM patients with a UCPCR > 0.20 nmol/mmol, those with UCPCR ≤ 0.20 nmol/mmol had a lower BMI (22.8 (21.2–25.4) kg/m^2^ vs. 25.2 (23.5–28.0) kg/m^2^, *P* = 0.006), FCP (0.39 (0.26–0.61) nmol/L vs. 0.66 (0.47–0.96) nmol/L, *P* < 0.001), PCP (0.93 (0.58–1.23) nmol/L vs. 1.55 (0.97–2.13) nmol/L, *P* < 0.001), and UA (308 (235–370) *μ*mol/L vs. 372 (302–436) *μ*mol/L, *P* = 0.011) and a higher proportion of insulin or secretagogue therapy (23/25 (92.0%) vs. 66/111 (59.5%), *P* = 0.002). Characteristics of the two patients who had a UCPCR < 0.20 nmol/mmol and who were using oral hypoglycemic agents are presented in Table 3. The ratios of male, insulin treatment, and complications and the levels of TC, TG, HDL-c, LDL-c, FBG, and A1C were similar in the two groups.

## 4. Discussion

### 4.1. Main Findings

Our study showed that UCPCR is a noninvasive tool that can be used to distinguish between T1DM and non-T1DM (T2DM and monogenic diabetes). UCPCR can distinguish between monogenic diabetes patients who need further genetic testing and who likely need noninsulin treatment and T1DM patients. However, in this study, UCPCR was not useful in distinguishing between monogenic diabetes and T2DM. Another important finding in our study is that the cut-off (≤0.20 nmol/mmol) for a differential diagnosis of diabetes is also helpful in identifying patients who need insulin or secretagogue therapy added to their treatment regimen to achieve their goal of glucose control. To the best of our knowledge, this is the first report on UCPCR in East Asia.

### 4.2. UCPCR Differs between Patients with T1DM, T2DM, and Healthy Subjects

In our study, UCPCR was lower in T1DM compared with T2DM or monogenic diabetes patients and healthy subjects. On the one hand, Besser et al. and Sebahat et al. demonstrated similar postprandial UCPCR results in T1DM, T2DM, and MODY subjects [15–17]. On the other hand, FCP in the T1DM group was also lower than that in T2DM and monogenic diabetes. Thus, we ascribed the lower UCPCR in T1DM patients to their absolute insulin deficiency pathogenesis.

UCPCR in monogenic diabetes was similar to that of healthy subjects in this study, but considering hyperglycemia in monogenic diabetes patients, we can speculate that there were relatively more dysfunctional beta cells in monogenic diabetes patients compared with healthy controls and that more sensitive methods such as a glucagon stimulation test would reveal these results. Additionally, patients who were taking secretagogues may confound this result, and the small sample size in our study might be another explanation.

### 4.3. The Advantages of UCPCR in the Evaluation of Beta Cell Function

Both UCPCR and serum C-peptide can be used to assess pancreatic beta cell function even with insulin microsecretors [18]. Compared with serum C-peptide, which should be separated from the serum via centrifugation and subsequently frozen to avoid protease hydrolysis, UCPCR was more convenient [11]. UCP is still stable even if it stays at room temperature for 3 days in boric acid [14] or if it is frozen at −80°C for 4 months (we tested 20 samples in an unpublished study). The stability and noninvasiveness of UCPCR can potentially facilitate many other experiments.

### 4.4. The Performance of UCPCR in the Differential Diagnosis of Different Diabetes Subtypes

UCPCR has been reported widely for measurement of endogenous insulin secretion and to distinguish between diabetes subtypes [15–17, 19]. Sebahat et al. reported that postprandial UCPCR ≥ 0.22 nmol/mmol could distinguish MODY from T1DM in children with 96.3% sensitivity and 85.7% specificity [17]. The cut-off point was similar to our result, which seemed more specific but less sensitive. Besser et al. performed several studies on UCPCR. They also demonstrated that UCPCR ≥ 0.20 nmol/mmol distinguished between MODY1 or MODY3 and T1DM with a sensitivity of 97% and a specificity of 96% in long-term adult diabetes patients (diabetes duration > 5 years) [15]. An absolute insulin deficiency appeared in most T1DM patients with a disease duration of more than 5 years [20], and therefore, the above-mentioned reports, which included mainly long-term T1DM patients (diabetes duration, 6.5 years (median, our study); 5.8 ± 3.3 years (mean ± SD, Sebahat et al.'s study); >5 years (Besser et al.'s study)), had similar optimal UCPCR cut-off levels. When a diabetes duration > 2 years is taken into consideration, the cut-off for the postprandial UCPCR level for typing non-T1DM (MODY, T2DM) and T1DM in children changed to 0.7 nmol/mmol (97% sensitivity and 96% specificity) [16].

UCPCR did not reliably distinguish between MODY and T2DM (AUC, 0.605) in our study, and Besser et al. reported a similar result [16]. This may be because a rapid decline in pancreas beta cell function is infrequent in both T2DM and monogenic diabetes.

### 4.5. UCPCR in Guiding the Individualized Treatments of T2DM Patients

In clinical practice, FCP is an important index for endocrinologists to use in deciding upon different therapies. There was already some evidence about the validity of C-peptide in predicting the time and intensity of insulin treatment [21, 22]. A stimulated C-peptide concentration ≤ 0.20 nmol/L may be a signal of absolute insulin deficiency and the likely requirement for future insulin treatment or even a predictor of intensive therapy [23]. Hope et al. reported that the urinary C-peptide creatinine ratio > 0.20 nmol/mmol in a mixed-meal tolerance test was a reliable indicator of retained endogenous insulin secretion [19].

In our study, 25/136 (18.4%) T2DM patients had a UCPCR ≤ 0.20 nmol/mmol. Hope et al. reported that 11/191 (5.8%) T2DM patients had UCPCR ≤ 0.20 nmol/mmol in a mixed-meal tolerance test. The patients in our study and Hope et al.'s study with UCPCR ≤ 0.20 nmol/mmol had a similar duration of disease as patients in our study (9.5 vs. 12 years), but the patients in our study were thinner (22.8 vs. 25.1 kg/m^2^).

In this study, most T2DM patients who had UCPCR ≤ 0.20 nmol/mmol (92.0%) were treated with insulin or secretagogues. They also had lower FCP and PCP compared with the patients with UCPCR > 0.20 nmol/mmol, which indicates a more deranged beta cell function in these patients. Two of 25 (8.0%) patients who had a UCPCR ≤ 0.20 nmol/mmol were treated without insulin or secretagogues, and both of these patients did not show signs of insulin resistance. These patients may need insulin or secretagogues in the future when their glucose control worsens because their hyperglycemia is likely caused by absolute insulin deficiency rather than insulin resistance. A recent study on the relationship between different T2DM diabetes subgroups and outcomes showed that the severely insulin-deficient subgroup had the highest risk of retinopathy [24]. Thus, UCPCR is very helpful for identifying patients who have poor glucose control and need early addition of insulin or secretagogues to their treatment regimen.

### 4.6. Limitations

To distinguish T1DM from T2DM with inadequate glycemic control might be problematic since glucotoxicity might cause suppressed insulin and C-peptide. There was a possibility that T2DM patients with very low UCPCR were composed of a few T1DM patients with negative islet antibodies. In this case, repeating UCPCR measurements in these patients when good glycemic control is achieved is necessary. Our study was a single-center study with relatively small sample size. Further studies are necessary to extend the validity of our findings.

## 5. Conclusion

The UCPCR is a practical noninvasive marker for the identification of TIDM from T2DM or monogenic diabetes which needs further gene diagnosis. The UCPCR ≤ 0.20 nmol/mmol could reflect severely impaired beta cell function and a need for insulin or secretagogues in T2DM patients.

## Figures and Tables

**Table 1:** Characteristics of 56 subjects with T1DM, 136 subjects with T2DM, 23 subjects with monogenic diabetes, and 59 healthy controls. All subjects were from the Beijing area, China.

	T1DM (*n* = 56)	T2DM (*n* = 136)	Monogenic diabetes (*n* = 23)	Healthy subjects (*n* = 59)	*P* value T1DM vs. T2DM	*P* value T1DM vs. monogenic diabetes	*P* value T2DM vs. monogenic diabetes
Male *n* (%)	28 (50.0)	87 (64.0)^✡^	13 (56.5)^✡^	26 (44.1)	0.073	0.549	0.494
Diagnosis age (yrs)	32.0 (23.3-46.0)	42.0 (32.0-49.0)	31.0 (23.0-34.5)	—	0.009^∗^	0.160	<0.001^∗^
Age (yrs)	46.0 (26.5-59.5)^✡^	53.0 (42.0-60.0)^✡^	35.0 (30.0-47.0)	36.0 (30.0-42.0)	0.031^∗^	0.148	0.001^∗^
Diabetes duration (yrs)	6.5 (1.5-13.0)	8.0 (2.3-14.0)	5.0 (1.0-16.0)	—	0.630	0.453	0.318
BMI (kg/m^2^)	22.3 (19.1-24.5)	24.9 (22.8-27.7)^✡^	22.2 (20.4-25.7)	22.0 (21.2-22.7)	<0.001^∗^	0.576	0.002^∗^
Parent affected *n* (%)	16 (28.6)	72 (52.9)	19 (90.5)	—	0.002^∗^	<0.001^∗^	0.001^∗^
TG (mmol/L)	0.98 (0.66-1.46)^✡^	1.47 (1.18-2.38)^✡^	1.16 (0.81-1.82)^✡^	0.73 (0.61-0.87)	<0.001^∗^	0.207	0.037^∗^
TC (mmol/L)	4.63 (3.71-5.63)	4.49 (3.68-5.20)	4.25 (3.92-5.53)	4.30 (3.86-4.92)	0.252	0.687	0.760
HDL-c (mmol/L)							
Male	1.29 (1.04-1.55)	0.97 (0.85-1.16)^✡^	1.06 (0.76-1.18)^✡^	1.25 (1.06-1.47)	0.002^∗^	0.010^∗^	0.786
Female	1.45 (1.23-1.67)	0.98 (0.80-1.15)^✡^	1.24 (1.00-1.49)	1.29 (1.16-1.64)	<0.001^∗^	0.043^∗^	0.236
LDL-c (mmol/L)	2.42 (1.69-3.47)	2.52 (1.97-3.23)	2.59 (2.33-3.22)	2.39 (1.78-2.75)	0.490	0.266	0.448
UA (*μ*mol/L)	280 (231-327)^✡^	357 (292-425)^✡^	310 (260-350)^✡^	238 (207-280)	<0.001^∗^	0.599	0.007^∗^
FBG (mmol/L)	9.8 (6.5-14.6)^✡^	7.2 (5.6-9.4)^✡^	8.1 (6.0-9.6)^✡^	5.1 (4.8-5.4)	<0.001^∗^	0.003^∗^	0.592
A1C (%)	9.2 (7.8-11.3)^✡^	9.1 (7.5-11.0)^✡^	8.2 (6.8-11.9)^✡^	5.2 (4.9-5.3)	0.367	0.224	0.659
FCP (nmol/L)	0.02 (0.01-0.10)	0.61 (0.41-0.91)	0.47 (0.27-0.76)	—	<0.001^∗^	<0.001^∗^	0.091
PCP (nmol/L)	0.05 (0.01-0.15)	0.98 (0.53-1.90)	0.89 (0.24-2.39)^†^	—	<0.001^∗^	<0.001^∗^	0.836
CRE (*μ*mol/L)	58.5 (47.0-68.8)	63.0 (52.3-72.0)^✡^	62.0 (56.0-84.0)^✡^	58.0 (44.0-68.7)	0.107	0.152	0.568
UCPCR (nmol/mmol)	0.03 (0.01-0.10)^✡^	0.47 (0.23-1.01)^✡^	0.67 (0.26-1.45)	0.71 (0.40-1.08)	<0.001^∗^	<0.001^∗^	0.107
Treatment *n* (%)							
Without insulin	0 (0.0)	79 (58.1)	4 (22.2)^†^	—	<0.001^∗^	0.003^∗^	0.004^∗^
Insulin±OHA	56 (100.0)	57 (41.9)	14 (77.8)^†^	—	<0.001^∗^	0.011^∗^	0.004^∗^

Data is median (interquartile range), unless otherwise stated. BMI: body mass index; CRE: creatinine; TG: triglyceride; TC: total cholesterol; HDL-c: high-density lipoprotein-cholesterol; LDL-c: low-density lipoprotein-cholesterol; UA: uric acid; FBG: fasting blood glucose; A1C: HbA1c; FCP: fasting C-peptide; PCP: postprandial C-peptide; OHA: oral hypoglycemic agent. ^∗^Statistical significance; ^✡^statistical significance between this diabetes group and healthy subjects; ^†^missing data: treatment for monogenic diabetes (*n* = 5), PCP for monogenic diabetes (*n* = 15).

**Table 2:** Clinical characteristics of T2DM patients with UCPCR ≤ 0.2 nmol/mmol vs. T2DM patients with UCPCR > 0.2 nmol/mmol.

	UCPCR ≤ 0.2 nmol/mmol *n* = 25	UCPCR > 0.2 nmol/mmol *n* = 111	*P* value
Male *n* (%)	18 (72.0)	69 (62.2)	0.355
Diagnosis age (yrs)	42.5 (33.0-48.5)	42.0 (32.0-49.0)	0.838
Age (yrs)	56.0 (41.5-61.0)	53.0 (42.0-60.0)	0.548
Diabetes duration (yrs)	9.5 (4.0-14.3)	7.0 (2.0-14.0)	0.365
BMI (kg/m^2^)	22.8 (21.2-25.4)	25.2 (23.5-28.0)	0.006^∗^
SBP (mmHg)	126 (120-140)	129 (120-138)	0.785
DBP (mmHg)	80 (70-80)	76 (68-80)	0.435
WL (cm)			
Male	92 (79-98)	95 (88-98)	0.066
Female	83 (78-90)	89 (81-97)	0.370
TG (mmol/L)	1.28 (1.07-2.12)	1.55 (1.21-2.58)	0.111
TC (mmol/L)	4.81 (3.74-5.66)	4.40 (3.68-5.09)	0.064
HDL-c (mmol/L)			
Male	1.03 (0.92-1.16)	0.96 (0.85-1.16)	0.812
Female	1.01 (0.73-1.20)	0.96 (0.81-1.14)	0.725
LDL-c (mmol/L)	2.81 (2.01-3.60)	2.49 (1.94-3.11)	0.371
UA (*μ*mol/L)	308 (235-370)	372 (302-436)	0.011^∗^
FBG (mmol/L)	7.4 (5.7-9.6)	7.1 (5.6-9.4)	0.606
FCP (nmol/L)	0.39 (0.26-0.61)	0.66 (0.47-0.96)	<0.001^∗^
PCP (nmol/L)	0.93 (0.58-1.23)	1.55 (0.97-2.13)	<0.001^∗^
UCPCR (nmol/mmol)	0.11 (0.08-0.15)	0.57 (0.37-1.14)	<0.001^∗^
A1C (%)	9.7 (7.5-12.0)	9.1 (7.5-10.9)	0.294
Complications			
DN *n* (%)	0	10 (9.0)	0.256
DR *n* (%)	5 (20.0)	15 (13.5)	0.607
DPN *n* (%)	9 (36.0)	35 (31.5)	0.666
Treatment *n* (%)			
Insulin/secretagogues	23 (92.0)	66 (59.5)	0.002^∗^

Data is median (interquartile range), unless otherwise stated. BMI: body mass index; SBP: systolic blood pressure; DBP: diastolic blood pressure; WL: waistline; TG: triglyceride; TC: total cholesterol; HDL-c: high-density lipoprotein-cholesterol; LDL-c: low-density lipoprotein-cholesterol; UA: uric acid; FBG: fasting blood glucose; FCP: fasting C-peptide; PCP: postprandial C-peptide; A1C: HbA1c; DN: diabetic nephropathy; DR: diabetic retinopathy; DPN: diabetic peripheral neuropath. ^∗^Statistical significance.

**Table 3:** The characters of the two patients who had a UCPCR < 0.20 nmol/mmol and without insulin or secretagogue treatment.

	Patient 1	Patent 2
Sex	Male	Female
Diagnosis age (yrs)	29	60
Age (yrs)	29	73
Diabetes duration (yrs)	0.6	13
BMI (kg/m^2^)	21.9	19.5
SBP (mmHg)	96	135
DBP (mmHg)	60	70
WL (cm)	68	78
TG (mmol/L)	2.93	0.61
TC (mmol/L)	6.4	5.69
HDL-c (mmol/L)	1.10	1.92
LDL-c (mmol/L)	3.90	2.73
UA (*μ*mol/L)	292	194
FBG (mmol/L)	9.4	5.13
FCP (nmol/L)	0.60	0.3
PCP (nmol/L)	0.71	1.77
FINS (*μ*U/ml)	2.72	31.18
PINS (*μ*U/ml)	5.92	8.20
UCPCR (nmol/mmol)	0.11	0.11
A1C (%)	13.5	6.6
Complications		
DN *n* (%)	—	—
DR *n* (%)	—	—
DPN *n* (%)	+	—
Treatment *n* (%)		
	Metformin 500 mg t.i.d.	Metformin 250 mg t.i.d.
	Acarbose tablet 50 mg t.i.d.	Acarbose tablet 50 mg t.i.d.

Data is median (interquartile range), unless otherwise stated. BMI: body mass index; SBP: systolic blood pressure; DBP: diastolic blood pressure; WL: waistline; TG: triglyceride; TC: total cholesterol; HDL-c: high-density lipoprotein-cholesterol; LDL-c: low-density lipoprotein-cholesterol; UA: uric acid; FBG: fasting blood glucose; FCP: fasting C-peptide; PCP: postprandial C-peptide; A1C: HbA1c; DN: diabetic nephropathy; DR: diabetic retinopathy; DPN: diabetic peripheral neuropath; OHA: oral hypoglycemic agent. ^∗^Statistical significance.

## Data Availability

The datasets during and/or analyzed during the current study are available from the corresponding authors on reasonable request.
